# Anxiety responses and testing intentions among gay and bisexual men using an AI-powered HIV/STI risk assessment tool: a quasi-experimental study

**DOI:** 10.1186/s12889-025-25064-2

**Published:** 2025-11-18

**Authors:** Phyu Mon Latt, Nyi Nyi Soe, Xianglong Xu, Yining Bao, David Lee, Jason J. Ong, Eric P. F. Chow, Lei Zhang, Christopher K. Fairley

**Affiliations:** 1 Artificial Intelligence and Modelling in Epidemiology Program, Melbourne Sexual Health Centre, Alfred Health, Melbourne, Australia; 2 School of Translational Medicine, Faculty of Medicine, Nursing and Health Sciences, Monash University, Melbourne, Australia; 3 School of Public Health, Shanghai University of Traditional Chinese Medicine, Shanghai, China; 4 China-Australia Joint Research Centre for Infectious Diseases, School of Public Health, Xi’an Jiaotong University Health Science Centre, Xi’an, Shaanxi Province People’s Republic of China; 5 Melbourne Sexual Health Centre, Alfred Health, Melbourne, Australia; 6 Department of Clinical Research, London School of Hygiene and Tropical Medicine, London, UK; 7 Centre for Epidemiology and Biostatistics, Melbourne School of Population and Global Health, The University of Melbourne, Melbourne, Australia; 8 Phase I Clinical Trial Research Ward, The Second Affiliated Hospital of Xi’an Jiaotong University, No.157 Xi Wu Road, Xi’an, Shaanxi Province 710004 China

**Keywords:** Anxiety, Artificial Intelligence, Risk assessment, Sexually transmittedinfections, Digital Health, STAI-6

## Abstract

**Introduction:**

Web-based tools for the assessment of the risk of sexually transmitted infections (STI) may increase testing but also may induce anxiety. We evaluated anxiety responses and testing intentions among gay, bisexual and other men who have sex with men (GBMSM) using *MySTIRisk*, an artificial intelligence-powered STI risk assessment tool, compared to a standard sexual health information webpage for GBMSMs.

**Methods:**

We conducted a quasi-experimental pre-post study at the Melbourne Sexual Health Centre between April and October 2024. Participants were allocated to either *MySTIRisk*, which provides personalised HIV/STI risk assessment, or a standard webpage providing general sexual health information for GBMSM on alternating days. We measured anxiety using the State-Trait Anxiety Inventory (STAI-6), which ranges from 6 to 24, before and after participants viewed their assigned websites. We defined clinically significant anxiety changes as ≥ 3 points on STAI-6. We used multivariable ordinal logistic regression analysis to evaluate clinically significant anxiety changes while controlling for baseline scores and demographic variables.

**Results:**

Our study population had a median age of 34 years (IQR: 28–42); 48% were born in Australia, and 69.7% had tertiary education. The baseline characteristics were similar between *MySTIRisk* (*n* = 150) and control (*n* = 150) groups. In the *MySTIRisk* group, STAI-6 scores increased significantly from baseline (median [IQR]: 10 [8, 12]) to post-intervention (11 [8, 13], *P* = 0.001). The control group showed a decrease from baseline (11 [9, 12]) to post-intervention (10 [8, 13], *P* = 0.01). Despite increased anxiety, *MySTIRisk* users maintained high user acceptability (92.0%) with similar testing intentions between groups (87.3% vs. 82.7%, *P* = 0.6). The multivariable ordinal logistic regression analysis showed that the intervention’s effect on anxiety was independent of demographic characteristics, except for employment status.

**Conclusions:**

While *MySTIRisk* increased anxiety in some users, it maintained high acceptability and did not deter testing intentions. These findings support implementing such tools with appropriate anxiety management strategies.

**Supplementary Information:**

The online version contains supplementary material available at 10.1186/s12889-025-25064-2.

## Introduction

Sexually transmitted infections (STIs) pose a significant global health concern, with over a million new cases daily and an estimated 374 million curable infections occurring annually [1]. Australia mirrors this global trend, experiencing a substantial increase in STI rates over the past decade. Australian notification data shows a threefold rise in syphilis diagnoses, a doubling of gonorrhoea diagnoses, and a 5.6% increase in chlamydia infections between 2013 and 2022, with gay and bisexual men being disproportionately affected by these increases [2].

The management of STIs is complex, particularly due to their often asymptomatic nature, leading to underdiagnosis and ongoing transmission [1]. Untreated STIs can contribute to complications such as infertility and increased cancer risk. Additionally, rectal gonorrhoea and chlamydia infections can increase the risk of HIV acquisition, particularly among men who have sex with men [3]. Consequently, accessible and effective screening tools are crucial for early diagnosis and treatment of HIV/STIs, aiming to mitigate individual and public health risks.

In response to this challenge, the Melbourne Sexual Health Centre (MSHC) developed a web-based and artificial intelligence (AI)-powered tool that assesses the risk of HIV, syphilis, chlamydia and gonorrhoea [4–7]. *MySTIRisk* differs from standard sexual health information webpages by providing personalised risk assessments tailored to individual users’ demographics and sexual practices. Whilst standard webpages offer general information applicable to all users, *MySTIRisk* provides individualised risk assessment for STIs based on each person’s unique profile. This personalised approach can enhance user engagement by providing direct relevance to their health behaviours [6]. However, while such tools offer promise in promoting early detection, they may inadvertently induce anxiety in users when confronted with their risk status [8].

Addressing the potential anxiety induced by these tools is crucial. Anxiety can deter individuals from seeking necessary medical care, thereby undermining the benefits of early detection and intervention [8–12]. Existing literature suggests that web-based HIV/STI risk assessment tools can have psychological impacts on users. For instance, one qualitative study that interviewed 32 GBMSMs in Boston, Massachusetts, in 2013–2014 found that some participants believed that an HIV risk prediction tool could elicit feelings of anxiety or fear in users [13].

While the *MySTIRisk* website is currently accessible online free of charge [4], we have yet to formally promote or integrate it into MSHC’s website. Before taking these steps, we want to assess the potential anxiety caused by the tool. Addressing this concern is essential to ensure that the benefits of early detection and intervention are not overshadowed by unintended emotional distress and to identify opportunities for refining the tool to maximise its effectiveness while minimising potential negative psychological impacts.

Our study seeks to understand the emotional impact of using *MySTIRisk* compared to a standard sexual health information webpage for men who have sex with men and how this may influence users’ intentions to seek testing. Specifically, we hypothesise that while *MySTIRisk* may increase anxiety levels in some users, it will also enhance their intentions to seek testing for HIV/STIs [6, 14]. By investigating this issue, we hope to optimise our web-based HIV/STI risk assessment tools, ensuring they are both safe and effective.

## Methods

### Study design

We conducted a quasi-experimental pre-post study following the Transparent Reporting of Evaluations with Non-randomised Designs (TREND) statement guidelines to evaluate and compare the impact of a web-based HIV/STI risk assessment tool (*MySTIRisk*) [4] against a standard clinic webpage providing sexual health information [15] on user anxiety and testing intentions between April and October 2024.

### Study population and recruitment

Our study included gay, bisexual, and other men who have sex with men (GBMSM) aged 18 years or older. We identified potential participants through daily queries of data collected via Computer-Assisted Self Interview (CASI) administered upon arrival at MSHC. CASI has been used at MSHC since 2008 as part of routine clinical care for obtaining sexual history and identifying patients’ risk factors for sexually transmitted infections, with an option for patients to consent to being contacted for research purposes. MSHC is a comprehensive sexual health clinic offering HIV and STI testing, treatment, prevention services, and sexual health consultations. Most participants in our study attended the clinic for routine HIV/STI screening or sexual health check-ups. The initial CASI screening identified individuals who were 18 years or older, assigned male at birth, reported having sex with men within the past 12 months, and had consented to receive SMS communications from MSHC. Eligible individuals received a single SMS invitation on the day after their MSHC visit. This SMS included a link to an anonymous online survey, which further screened participants based on current gender identity and sexual orientation.

We used the Qualtrics online survey platform (Qualtrics, Provo, UT) to administer the survey. Participants were introduced with a brief study description and a link to a plain-language statement outlining the study. They were then asked to consent by selecting either the “Agree” or “Disagree” option. This step was necessary to proceed with the survey. If a participant clicked “Disagree,” the survey would end immediately. After they consented, participants underwent a second screening process to confirm eligibility. Final inclusion criteria were: age 18 years or older, assigned male at birth, sexually active within the past 12 months, identify as GBMSM and do not have high anxiety scores (≤ 14 on the Spielberger State-Trait Anxiety Inventory (STAI-6) scale). We excluded participants who reported high anxiety levels with probable clinical anxiety in the first STAI-6 survey to protect their wellbeing [16]. The survey automatically ended for these individuals, and they were immediately provided with counselling resources and support services free of charge.

### Survey content and procedures

All study procedures were completed in a single, continuous online session immediately after participants provided consent and confirmed eligibility. The surveys comprised three main parts: pre-website questions, website interaction, and post-website questions. In the pre-website section, participants answered demographic questions, including age, country of birth, education level, and employment status. They also provided information on their HIV/STI testing history and perceived risk. The STAI-6 was administered to assess baseline anxiety levels. Participants did not know their assigned group until after the baseline anxiety inventory was completed.

We used an alternating-day assignment method for group allocation. Clients attending MSHC on the first day of recruitment were assigned to the *MySTIRisk* group and those on the second day to the control group, and this alternating pattern continued throughout the recruitment period. All participants received only one SMS invitation corresponding to their assigned group to prevent crossover.

After interacting with their assigned websites, participants completed the post-website survey. This included a repeat of the STAI-6 to measure changes in anxiety and questions about their likelihood of getting tested for HIV/STIs in the next three months. Specifically, participants were asked “How likely are you to get tested for HIV/STIs in the next three months?” with response options ranging from 1 (extremely unlikely) to 5 (extremely likely), as well as an option for “unsure/prefer not to answer. Participants who viewed *MySTIRisk* also provided feedback on the website’s usability, clarity of information, and its potential influence on their sexual behaviours.

Upon completing the survey, participants had the option to access an alternative website if they desired additional information, regardless of their initial group assignment. This option was provided for ethical reasons to ensure all participants had access to both types of health information.

### Intervention and control

*MySTIRisk* is a web and artificial intelligence (AI)-based tool developed by MSHC that assesses users’ risk for STIs, including HIV, syphilis, gonorrhoea and chlamydia, based on their reported demographic and sexual practices [4]. *MySTIRisk* calculates personalised risk scores using machine learning algorithms trained on data from MSHC attendees. The tool compares individual participant responses to this training dataset to generate risk predictions. It includes a series of questionnaires about demographic and sexual practices, an AI algorithm that calculates personalised risk scores, recommendations for testing and prevention, and a visual representation of the risk level (see Appendix A). The control group accessed MSHC’s standard sexual health information webpage for men who have sex with men [15], which contained general information about HIV and STI prevention, testing recommendations, safe sex practices, and educational resources without personalised risk assessment (see Appendix B). Upon survey completion, participants had the opportunity to enter a drawing to win a $50 gift voucher via a link to a separate survey.

### Sample size calculation

We recruited 300 participants, with equal allocation to the *MySTIRisk* and control groups. We calculated the sample size to detect a clinically meaningful change in STAI-6 anxiety scores before and after using the *MySTIRisk* website. Prior studies using the STAI-20 scale suggested that a 10-point difference can be considered clinically meaningful [17, 18]. For the 6-item STAI-6 short form, we estimated a corresponding Minimal Clinically Important Difference (MCID) of 3 points. To detect a 3-point change in mean STAI-6 scores before and after using *MySTIRisk*, with a power of 90%, a standard deviation of 8 [19], and a significance level of 0.05, we calculated the sample size of 150 participants for each group. We did not factor in attrition, as participants would complete the study in a single session. Recruitment for each group was stopped when it reached the target sample size of 150 participants, ensuring our pre-specified sample size was achieved.

### Anxiety assessment

We used the six-item short form of the Spielberger State-Trait Anxiety Inventory (STAI-6), a validated, shortened version of the original 20-item STAI-State scale [20] (Table S1). The STAI-6 consists of six questions, each rated on a 4-point Likert scale ranging from 1 (Not at all) to 4 (Very much). Three of the items are reverse-scored (“calm,” “relaxed,” “content”). The STAI-6 is a practical choice for this study due to its brevity, taking only a few minutes to complete.

### Statistical analysis

We used STATA (version 17, StataCorp) for all data analyses in this study. Our final analysis included all participants who completed both the pre-website and post-website anxiety assessments. As our study design collected all data in a single session, we excluded those who did not complete both assessments. The complete questionnaires used in this study for both the intervention and control websites are available as Supplementary Files 1 and 2. For descriptive statistics, we presented age as the median (interquartile range) due to its non-normal distribution and categorical variables as frequencies and percentages. We compared baseline characteristics between intervention and control groups using the Mann-Whitney U test for age and chi-square tests for categorical variables.

Our Qualtrics survey included a ‘Prefer not to answer’ option for all questions, which allowed participants to express discomfort without skipping questions entirely. We included the ‘Prefer not to answer’ responses as a distinct category in our descriptive analyses to maintain transparency and accurately reflect participant choices.

For our primary outcome of anxiety scores, we used the Wilcoxon signed-rank test to assess changes within groups and the Mann-Whitney U test to compare changes between groups due to the non-normal distribution of the anxiety scores. We used the Shapiro-Wilk test to assess the normality and the Variance Ratio Test (F-test for equality of variances) for homoscedasticity. To determine the magnitude of the intervention effect, we calculated Cohen’s d test, classifying them as small (0.20 ≤ d < 0.50), medium (0.50 ≤ d < 0.80), or large (d ≥ 0.80) [21].

We used the Minimal Clinically Important Difference (MCID) to assess clinically significant changes. For the STAI-6, we defined the MCID as a change of 3 points [17, 18]. We categorised participants as having increased, decreased, or unchanged anxiety based on this threshold. We then compared the proportions of participants in each category between groups using chi-square tests.

To examine demographic influences on anxiety changes, we conducted a multivariable ordinal logistic regression analysis using the MCID categories (decreased, unchanged, increased) as the outcome. We first performed univariable ordinal logistic regression for potential predictors. Variables showing associations at *P* < 0.20 (age, country of birth, employment status, and perceived HIV/STI risk) and theoretically important variables (group allocation and baseline anxiety) were included in the initial model. We used backward stepwise elimination with a removal threshold of *P* > 0.05. Missing data were minimal (< 5%), and we used complete case analysis to handle these missing values. We assessed the appropriateness of ordinal logistic regression by testing key assumptions. The parallel lines test confirmed the assumption of proportional odds. The model specification was evaluated using link tests, and variance inflation factors were examined to assess multicollinearity among predictors.

For the secondary outcome of testing intentions after using *MySTIRisk* or viewing the control webpage, we used chi-square tests to compare intentions between groups. Response categories were recoded as follows: ‘Unlikely’ (extremely unlikely and somewhat unlikely), ‘Neutral’ (neither likely nor unlikely), and ‘Likely’ (somewhat likely and extremely likely), while ‘Unsure/Prefer not to answer’ remained unchanged. We also analysed *MySTIRisk* website feedback using descriptive statistics for those in the intervention group.

## Results

### Participant characteristics

**Fig. 1:**
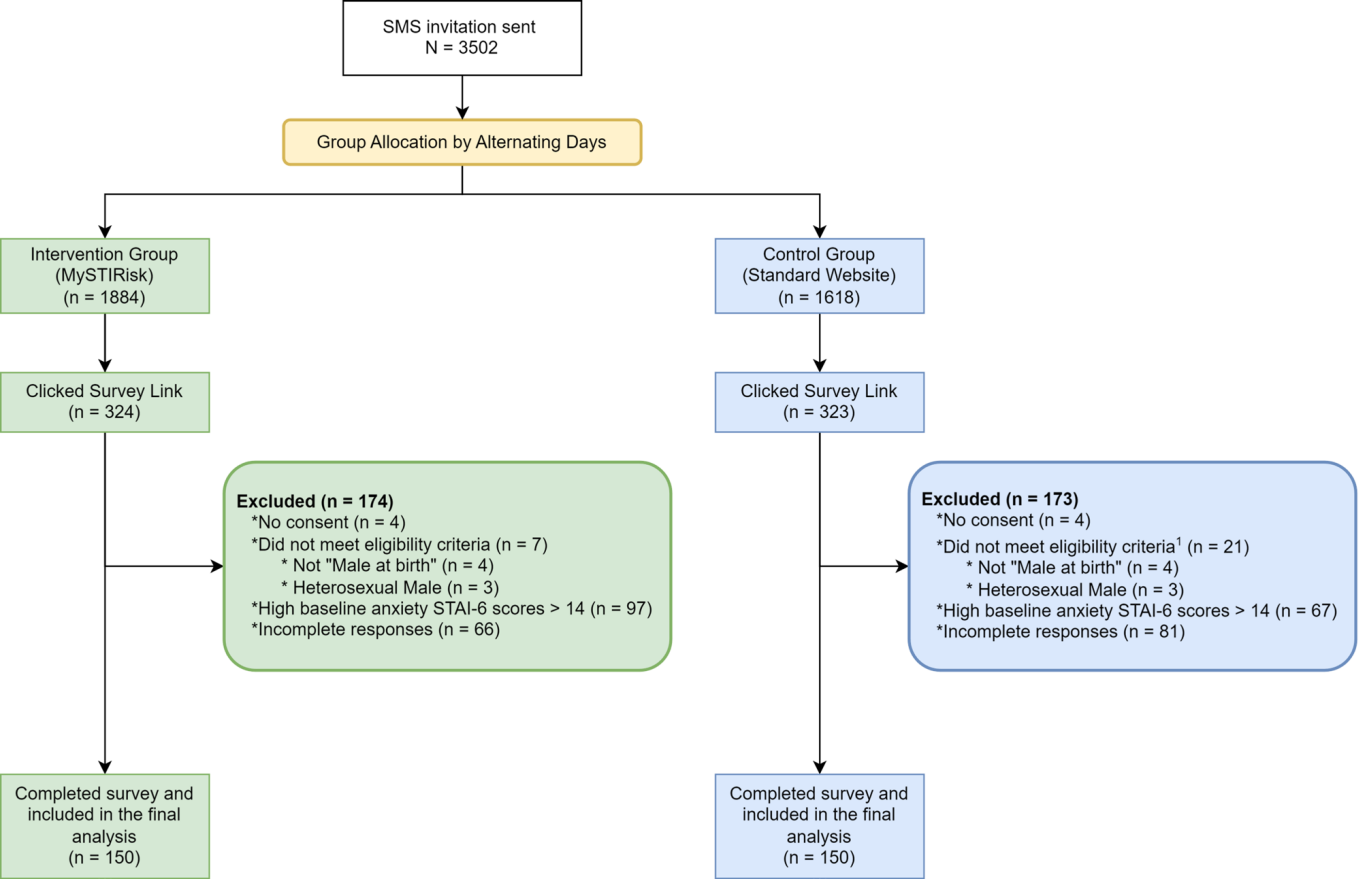
Participant Flow Diagram

We sent SMS invitations to 3,502 eligible individuals who had consented to receive messages from MSHC. Of those who accessed our survey (*n* = 647), we excluded individuals who did not consent (*n* = 8), those who did not meet eligibility criteria (*n* = 28), those with high baseline anxiety (STAI-6 score > 14) (*n* = 164), and those with incomplete responses (*n* = 147). Our final sample consisted of 300 gay, bisexual and other men who have sex with men (GBMSM), with 150 participants in each group (see Fig. 1 for detailed participant flow).

The baseline characteristics of the *MySTIRisk* and control groups were similar (*P* > 0.1) for age (median 34 years), country of birth in Australia (MySTIRisk vs. control: 42.7% vs. 53.3%), tertiary education level (70.0% vs. 69.3%), and had tested for HIV/STI within 6 months (91.1% vs. 89.7%). Most participants in both groups reported low to medium self-perceived HIV/STI risk (see Table 1 for detailed characteristics).

**Table 1:** Demographic characteristics of study participants

	Total (*N* = 300)	Intervention Group MySTIRisk (*n* = 150)	Control Group Sexual Health Information Webpage (*n* = 150)	*P*-value
Age, years				
Median (IQR)	34 (28–42)	34 (28–41)	34 (28–42)	0.9
Country of origin, n (%)				
Australia	144 (48.0)	64 (42.7)	80 (53.3)	0.1
Other country	155 (51.7)	85 (56.7)	70 (46.7)	
Not sure/Prefer not to answer	1 (0.3)	1 (0.7)	-	
Highest level of education, n (%)				
Postgraduate level	93 (31.0)	45 (30.0)	48 (32.0)	0.9
Bachelor level	116 (38.7)	60 (40.0)	56 (37.3)	
Diploma level	30 (10.0)	16 (10.7)	14 (9.3)	
Certificate level	25 (8.3)	11 (7.3)	14 (9.3)	
High school	35 (11.7)	17 (11.3)	18 (12.0)	
Primary school	1 (0.3)	1 (0.7)	-	
Employment status, n (%)				
Student	45 (15.0)	25 (16.7)	20 (13.3)	0.9
Full-time employment or self-employed	170 (56.7)	86 (57.3)	84 (56.0)	
Part-time/casual employment	55 (18.3)	26 (17.3)	29 (19.3)	
Retired	16 (5.3)	7 (4.7)	9 (6.0)	
Unemployed or not working	11 (3.7)	5 (3.3)	6 (4.0)	
Unable to work	2 (0.7)	1 (0.7)	1 (0.7)	
Other	1 (0.3)	-	1 (0.7)	
Previous HIV/STI Testing, n (%)				
Yes	291 (97.0)	146 (97.3)	145 (96.7)	0.7
No	9 (3.0)	4 (2.7)	5 (3.3)	
Last HIV/STI Test, n (%)				
Within the past 6 months	263 (90.4)	133 (91.1)	130 (89.7)	0.7
Within the past year	12 (4.1)	5 (3.4)	7 (4.8)	
1–2 years ago	10 (3.4)	6 (4.1)	4 (2.8)	
More than 2 years ago	6 (2.1)	2 (1.4)	4 (2.8)	
Self-Perceived STI Risk, n (%)				
High Risk	48 (16.0)	21 (14)	27 (18)	0.8
Medium Risk	108 (36.0)	55 (36.7)	53 (35.3)	
Low Risk	135 (45.0)	70 (46.7)	65 (43.3)	
Not sure/Prefer not to answer	9 (3.0)	4 (2.7)	5 (3.3)	

*IQR* Interquartile Range, *STI* sexually transmitted infection

### Baseline and changes in STAI-6 anxiety scores

Our initial baseline STAI-6 analysis included 530 participants who completed the pre-survey assessment. Of these, 164 participants had high anxiety scores (STAI-6 > 14), and 66 participants did not complete the post-survey assessment. The Mann-Whitney test showed no significant difference in baseline anxiety scores between groups (*P* = 0.9) (Figure S1). Comparison of the baseline characteristics between participants with STAI-6 ≤ 14 (*n* = 366) and > 14 (*n* = 164) showed similar patterns in HIV/STI testing recency (*P* = 0.2) and risk perception (*P* = 0.5), but those with scores of > 14 were less likely to have had an STI (Table S2).

The overall median baseline anxiety score among included participants was 10 [8, 13]. To ensure our group allocation method did not introduce systematic bias, we examined the distribution of participants between *MySTIRisk* and control groups across different days of the week. Our alternating-day assignment method showed no significant association between the day of the week and group allocation (χ² test, *P* = 0.7).

After interacting with their assigned websites, both groups showed significant changes in anxiety scores from baseline, but in opposite directions. The *MySTIRisk* group experienced a significant increase from baseline (10 [8, 12]) to post-intervention (11 [8, 13]; *P* = 0.001), while the control group showed a significant decrease from baseline (11 [9, 12]) to post-intervention (10 [8, 13]; *P* = 0.3) (see Table 2). Although baseline anxiety scores differed between groups (*P* = 0.04), the difference in the anxiety scores after the websites was not significantly different (*P* = 0.3) (Table 2). The effect size (Cohen’s d = 0.55) indicated a moderate effect of the intervention. We found no significant interaction between group assignment and baseline anxiety (*P* = 0.2).

**Table 2:** Changes in STAI-6 scores between the two groups

Group	Baseline	After website	*P*-value** (Wilcoxon Signed-Rank Test)
	Median [IQR]	Median [IQR]	
MySTIRisk Group	10 [8, 12]	11 [8, 13]	0.001
Control Group	11 [9, 12]	10 [8, 13]	0.01
*P*-value** (Mann-Whitney U Test)	0.04	0.3	

*IQR* Interquartile Range

***P-values from non-parametric tests compare entire distributions rather than just medians

The distribution of STAI-6 scores (Figure S2) illustrates the trends visually. After website use, the *MySTIRisk* group showed a rightward shift in score distribution, with an increase in participants scoring in higher ranges (>13) and fewer in lower ranges (6–8). In contrast, the control group showed a leftward shift, with more participants scoring in lower ranges (6–7) post-exposure.

### Clinically significant changes in anxiety

We analysed clinically significant changes in anxiety levels using the MCID threshold of 3 points on the STAI-6 scale (Table 3). In the *MySTIRisk* group, 23.3% (*n* = 35) of participants experienced a clinically significant increase in anxiety, compared to 9.3% (*n* = 14) in the control group. Conversely, 5.3% (*n* = 8) of the *MySTIRisk* group and 14.0% (*n* = 21) of the control group showed a clinically significant decrease in anxiety. Most participants showed no clinically significant change (*MySTIRisk*: 71.3%, *n* = 107; Control: 76.7%, *n* = 115). Chi-square analysis revealed a significant difference in the distribution of these categories between groups (*P* = 0.001).

**Table 3:** Clinically significant changes in anxiety between the two groups

MCID Changed Category	MySTIRisk (*n* = 150)	Control (*n* = 150)	Total (*N* = 300)
Increased^a^, n (%)	35 (23.3)	14 (9.3)	49 (16.3)
No Change, n (%)	107 (71.3)	115 (76.7)	222 (74.0)
Decreased^a^, n (%)	8 (5.3)	21 (14.0)	29 (9.7)

Chi-square test: *P* < 0.001

^a^Change ≥ 3 points on STAI-6 scale

In multivariable ordinal logistic regression, after adjusting for baseline anxiety and employment status, participants using *MySTIRisk* had higher odds of experiencing clinically significant anxiety increases (adjusted OR: 3.12, 95% CI: 1.75–5.56, *P* < 0.001). Among demographic factors, only being out of the workforce was significantly associated with increased anxiety (adjusted OR: 4.84, 95% CI: 1.33–17.65, *P* = 0.02) (Table 4).

**Table 4:** Univariable and multivariable analysis of factors associated with clinically significant changes in anxiety (MCID)

Predictor	Crude OR (95% CI)	*P*-value	Adjusted OR* (95% CI)	*P*-value
Group				
Control	Ref		Ref	
MySTIRisk	3.10 (1.76–5.45)	< 0.001	3.12 (1.75–5.56)	< 0.001
Baseline STAI-6 scores	0.92 (0.83–1.02)	0.1	0.95 (0.86–1.06)	0.4
Age	0.99 (0.97–1.01)	0.4		
Country of origin				
Australia	Ref			
Other country	1.13 (0.67–1.88)	0.7		
Highest level of education				
Postgraduate level	Ref			
Bachelor level	0.92 (0.49–1.70)	0.8		
Diploma level	1.16 (0.47–2.90)	0.7		
Certificate level	1.04 (0.40–2.71)	0.9		
Primary/High school	1.28 (0.53–3.07)	0.6		
Employment Status				
Student	Ref		Ref	
Full-time employment or self-employed	0.99 (0.47–2.10)	0.9	0.95 (0.44–2.07)	0.9
Part-time/casual employment	0.67 (0.27–1.68)	0.4	0.79 (0.31–2.00.31.00)	0.6
Retired	0.97 (0.27–3.52)	0.9	0.91 (0.24–3.47)	0.9
Not in the workforce	4.19 (1.22–14.36)	0.02	4.84 (1.33–17.65)	0.02
Self-Perceived STI Risk				
High Risk	Ref			
Medium/Average Risk	1.64 (0.77–3.50)	0.2		
Low Risk	1.36 (0.65–2.84)	0.4		

*MCID* Minimal Clinically Important Difference defined as ≥3 point change in STAI-6 scores

Outcome categories: Decreased (≥3 point decrease), Unchanged (<3 point change), Increased (≥3 point increase)

*STI* sexually transmitted infection, *OR* Odds Ratios

*Adjusted OR from the final multivariable model after backward stepwise elimination (removal threshold *P* > 0.05), with the baseline STAI-6 scores included regardless of their significance level

### Testing intentions

Following exposure to their assigned websites, both groups reported increased intentions to seek HIV/STI testing within the next three months. In the *MySTIRisk* group, 87.3% (*n* = 131) indicated they were likely to get tested, compared to 82.7% (*n* = 124) in the control group. A small proportion remained neutral (4.0%, *n* = 6 in both groups) or unlikely to seek testing (*MySTIRisk*: 8.0%, *n* = 12; Control: 11.3%, *n* = 17). Chi-square analysis showed no significant difference in testing intentions between the groups (*P* = 0.6) (Table 5).

**Table 5:** Intention to test in the next three months between the two groups

Testing Intention	Total (*N* = 300)	MySTIRisk (*n* = 150)	Control (*n* = 150)	*P*-value*
Likely, n (%)	255 (85.0)	131 (87.3)	124 (82.7)	0.6
Neutral, n (%)	12 (4.0)	6 (4.0)	6 (4.0)	
Unlikely, n (%)	29 (9.7)	12 (8.0)	17 (11.3)	
Unsure/Prefer not to answer, n (%)	4 (1.3)	1 (0.7)	3 (2.0)	

*Chi-square test comparing the distribution of responses between groups

### MySTIRisk feedback

Most participants found *MySTIRisk* easy to use, with 92.0% (*n* = 138) reporting it was “somewhat easy” or “very easy” to use. The information provided was rated as clear and understandable by 90.7% (*n* = 136) of participants, with 60.0% finding it “very clear.” Regarding behavioural impact, 32.7% (*n* = 49) indicated that their *MySTIRisk* results would lead them to change their sexual behaviours, while 57.3% (*n* = 86) did not anticipate behaviour changes. Nearly three-quarters of participants (72.7%, *n* = 109) reported they would recommend *MySTIRisk* to friends or partners (Table S3).

## Discussion

Our study found that the AI-powered HIV/STI risk assessment tool, *MySTIRisk*, increased clinically significant anxiety levels in GBMSMs by about 14.0% more than a standard sexual health webpage (23.3% of intervention vs. 9.3% of control group, *P* = 0.001). Despite these increases in anxiety, the tool maintained high user acceptability (92% ease of use) and did not deter testing intentions (87.3% likely to test). These findings align with previous qualitative research by Gilkey et al., who found that HIV risk prediction tools could provoke anxiety among users, although they did not quantify these effects nor assess how long they lasted [13]. Our study extends this understanding by providing the first quantitative evidence of anxiety responses to an online HIV/STI risk assessment tool. Importantly, while the moderate effect size (Cohen’s d = 0.55) suggests meaningful anxiety increases, these did not translate into reduced testing intentions, contrasting with previous research showing anxiety as a barrier to healthcare-seeking [9, 22–24]. These findings have important implications for implementing digital HIV/STI risk assessment tools, suggesting that while such tools may increase user anxiety, this emotional response does not necessarily impede their primary purpose of encouraging HIV/STI testing. This balance between managing user anxiety and promoting testing behaviour represents a key consideration for the future development of digital sexual health interventions.

A key finding of our study was the differential impact on anxiety levels between *MySTIRisk* users and those exposed to standard sexual health information. While 9.3% of participants in the control group experienced increases in clinically significant anxiety, this proportion was significantly higher at 23.3% among *MySTIRisk* users. The additional 14.0% increase in clinically significant anxiety could be attributed to the personalised risk assessment provided by *MySTIRisk*. This finding also highlighted the potent psychological impact of individualised risk information, which appears to elicit stronger emotional responses than general health education [25]. However, a key issue when evaluating the impact of this increase in anxiety is how long it lasts and whether, as one might expect, it dissipates once testing has occurred. We were unable to find other published studies of the duration of similar digital interventions. For comparison, the anxiety induced by HIV testing lasts only hours to days and dissipates upon receiving a negative result [26]. A limitation of our single-session design is that we cannot determine how long the increased anxiety we observed persists or its impact on quality of life. Future research should investigate the duration of anxiety responses to HIV/STI risk assessment tools through longitudinal follow-up.

Building on our understanding of anxiety responses, we examined how these emotional changes influenced healthcare-seeking intentions among the participants. Despite the increased anxiety levels, 87.3% of *MySTIRisk* users reported they were likely to seek HIV/STI testing, comparable to the control group (82.7%). This finding reflects the complex relationship between anxiety and health-seeking behaviours. When we interpret these results alongside the anxiety data, contrary to our initial hypothesis, our findings reveal that while *MySTIRisk* significantly increased anxiety scores, this did not translate into significantly higher testing intentions compared to the control group (87.3% vs. 82.7%, *P* = 0.6). However, the moderate increase in anxiety scores observed in the *MySTIRisk* group was associated with maintained high testing intentions (87.3%), which is consistent with evidence from health behaviour theories that moderate anxiety levels can motivate rather than deter health screening behaviours, even though both interventions achieved similar motivation levels through different psychological pathway [27, 28]. This relationship requires careful interpretation given that both groups demonstrated similarly high testing intentions, and the control group showed decreased anxiety while maintaining comparable intentions. This pattern aligns with the Health Belief Model, where perceived risk can motivate health-seeking behaviours [29]. Goodwin et al.‘s systematic review further supported this concept, showing how anxiety often serves as a catalyst for cancer screening participation [30]. Similarly, several studies demonstrated how digital health interventions could help manage anxiety and promote proactive health behaviours [31–33]. However, these results should be considered in the context of our study population, GBMSMs, who have already accessed sexual health services and likely have high health literacy. Among participants with low to moderate baseline anxiety levels in our study, the relationship between increased anxiety and maintained testing intentions suggests that digital risk assessment tools may effectively balance emotional responses with beneficial health behaviours in this population, particularly among health-engaged individuals who regularly access sexual health services.

Additionally, our analysis revealed that participants who were out of the workforce (*n* = 13) showed greater anxiety increases compared to students. Although this finding should be interpreted with caution due to the small sample size, it suggests that employment status may influence responses to personalised risk information. This highlights the importance of considering socioeconomic factors when implementing digital health interventions, and further research with larger samples is needed to explore these relationships.

The findings of our study have several important implications for public health practice. While web-based HIV/STI risk assessment tools can maintain higher testing intentions, they must be designed and deployed in ways that minimise undue psychological distress. Healthcare services implementing such tools should consider incorporating real-time anxiety monitoring and support features, such as online counselling options or direct links to healthcare providers. The stronger emotional impact of personalised risk information compared to general health information highlights the importance of providing adequate support resources alongside risk assessment tools. Moreover, the varied anxiety responses among users suggest that a one-size-fits-all approach may not be appropriate. Instead, tailoring risk communication strategies to specific populations could enhance effectiveness while mitigating negative impacts.

Our study has several notable strengths and unique features. First, to our knowledge, this is the first study to specifically examine the anxiety-inducing potential of a web-based HIV/STI risk assessment tool for GBMSM. This focus addresses an important gap in the literature and provides valuable insights for developing and implementing similar tools. Second, the use of a quasi-experimental pre-post design with a control group allows for a robust evaluation of *MySTIRisk*’s impact on anxiety levels. Third, by conducting the study in a major sexual health clinic and including GBMSM who are individuals at higher risk, we enhanced the ecological validity of our findings and offered targeted insights that can inform tailored interventions. Fourth, we used validated measures, particularly the STAI-6 for anxiety assessment, and clinically meaningful thresholds to quantify anxiety responses, moving beyond the qualitative insights of previous research. Our sample size (*N* = 300) offered adequate statistical power to detect anxiety changes. Lastly, integrating appropriate statistical approaches, including ordinal logistic regression, helped control for baseline differences and potential confounders, strengthening our conclusions about the relationship between digital risk assessment tools and user anxiety.

However, our study has several important limitations. First, recruiting participants from a single urban sexual health centre may have biased our sample towards a more health-conscious GBMSM, potentially limiting generalisability. Additionally, since participants were recruited a day after their clinic visit, they may have had heightened baseline anxiety levels and testing intentions due to recent discussions about sexual behaviours with healthcare providers, which may not reflect responses in community-based settings. While we chose this approach for ethical reasons to minimise potential harm from anxiety-inducing information, future studies could explore broader populations using an improved website. Second, our focus on GBMSM, while limiting applicability to other groups, was intentional given their higher risk profile for HIV and STIs. Third, our reliance on self-reported measures, particularly testing intentions rather than actual behaviour, may be subject to social desirability bias, which we attempted to mitigate through validated scales and assured anonymity. Fourth, we excluded individuals with an anxiety score over 14 to avoid potential harm, which means we cannot determine the intervention’s effect in this group. While baseline anxiety levels are not routinely pre-screened in real-world implementation, it was reassuring that anxiety increased only in a small proportion of users, and this situational anxiety would likely last only until testing was completed. Our data suggest only marginal differences in STI risk and testing between those with and without baseline anxiety over 14, indicating that differential effects of the intervention between these groups are unlikely. Fifth, although our alternating-day allocation resulted in a small but significant difference in baseline anxiety scores between groups, we addressed this potential source of bias by using the Mann-Whitney test for distribution comparison and multivariable logistic regression to ensure the intervention effect was independent of baseline characteristics. Sixth, the single-session design suited our aim of measuring immediate anxiety responses but limited our ability to assess whether these anxiety changes persist over time or influence long-term testing behaviours. A transient increase in anxiety would be expected and may not have an impact on quality of life. Lastly, our concise survey design did not extensively explore specific anxiety-inducing aspects of *MySTIRisk*, suggesting the need for future qualitative studies to provide deeper insights into users’ experiences.

These findings also point to several key directions for future research. Longitudinal studies are needed to understand whether anxiety changes persist over time and how they influence actual testing behaviours rather than just intentions. Research should also examine which support features work best in managing user anxiety while maintaining the benefits of personalised risk assessment. Developing adaptive versions of *MySTIRisk* that cater to different anxiety profiles and health literacy levels could help extend its use beyond health-engaged GBMSM populations. Additionally, research should focus on creating clear implementation guidelines that balance the benefits of personalised risk information with user well-being. These insights will be crucial for informing broader digital health screening policies and improving future iterations of STI risk assessment tools.

## Supplementary Information

Supplementary Material 1.

Supplementary Material 2.

Supplementary Material 3.

Supplementary Material 4.

Supplementary Material 5.

## Data Availability

The data that support the findings of this study are not openly available due to reasons of sensitivity and are available from the corresponding author, Dr. Phyu Mon Latt, at phyu.latt@monash.edu, upon reasonable request. Data are in controlled access data storage at the Melbourne Sexual Health Centre.
