# Supplementary material for: Anxiety responses and testing intentions among gay and bisexual men using an AI-powered HIV/STI risk assessment tool: a quasi-experimental study
Source: BMC Public Health. 2025 Nov 18;25:4028. doi: 10.1186/s12889-025-25064-2 (PMC12625431; doi:10.1186/s12889-025-25064-2)
Supplement: Supplementary file 1 — Supplementary Material 1. [file 12889_2025_25064_MOESM1_ESM.pdf]

Part I

Introduction

Introduction

Thank you for your interest in participating in this research. You received a link to this survey via an SMS text message because you previously consented to getting health-related messages from the Melbourne Sexual Health Centre.

We developed a website for the public to measure their risk of having HIV and other sexually transmitted infections (STIs), which is called "*MySTIRisk*". We would like your help testing how this site makes you feel and how likely you are to have a test for these infections.

Half of the people will see the *MySTIRisk* website, and the other half will get access to information on the MSHC website that has sexual health information. To measure how the website makes you feel, we have a survey before and after so we can measure changes in anxiety levels. We will also collect some background information, including sexual risk.

It will take around 15 minutes of your time to complete the survey.

You do not have to participate in this survey if you don't want to. This survey will be anonymous and will not collect any information that can identify you. Your responses to this survey will remain confidential to the MSHC research team.

This study is approved by the Alfred Hospital Ethics Committee (741/23). Please read the participant information sheet by clicking HERE.

Do you agree to participate in the survey?

- ◯ I agree
- ◯ I disagree

Block 3

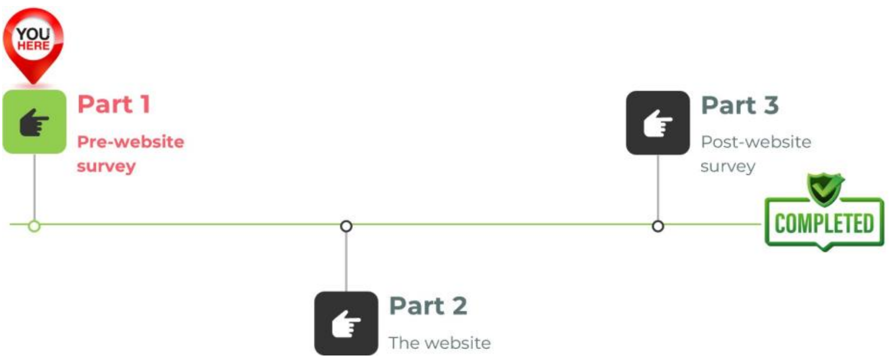

This survey has 3 parts:

Part 1: Pre-website Questions
In this first part, we ask you things about yourself and 6 questions about how you are feeling.

Part 2: The website

Next, you will either use the MySTIRisk website or view a standard sexual health webpage. Please read carefully. Please read carefully.

Part 3: Post-Website S
Finally, you'll answer questions about how the website made you feel.

Please answer honestly - we are interested in your opinions and feelings. Your responses are anonymous.

Demographic Questions

Part 1: Pre-website questions

What sex were you assigned at birth?

- ○ Male
- ○ Female
- ○ Intersex
- ○ Don’t know/ Prefer not to say

Do you consider yourself to be:

- ○ Lesbian / Gay / Homosexual
- ○ Bisexual
- ○ Straight/Heterosexual
- ○ Queer
- ○ [ ] Different identity, Please specify:
- ○ Prefer not to say

How old are you? (write number of years)

[ ]

Where were you born?

- ○ Australia
- ○ Other country
- ○ Not sure/Prefer not to answer

How long have you been in Australia?

- ○ Less than 1 year
- ○ Less than 5 years
- ○ 5 years or longer
- ○ Not sure/Prefer not to answer

What is the highest level of education you have completed?

- ○ Postgraduate level
- ○ Bachelor level
- ○ Diploma level
- ○ Certificate level
- ○ High school
- ○ Primary school
- ○ ______ Other (Please Specify)
- ○ Not sure/Prefer not to answer

What is your current employment status? (Tick all that apply]

- ○ Student
- ○ Full-time employment or self-employed
- ○ Part-time /casual employment
- ○ Retired
- ○ Unemployed or not working
- ○ Unable to work
- ○ ______ Other (Please specify)
- ○ Not sure/Prefer not to answer

Have you ever been tested for sexually transmissible infections such as HIV, syphilis, gonorrhoea or syphilis?

- ○ Yes
- ○ No
- ○ Not sure/Prefer not to answer

Have you ever been diagnosed with sexually transmissible infections such as HIV, syphilis, gonorrhoea or syphilis?

- ○ Yes
- ○ No
- ○ Not sure/Prefer not to answer

When was your last HIV/STI test?

- ○ Within the past 6 months
- ○ Within the past year
- ○ 1-2 years ago
- ○ More than 2 years ago
- ○ Never tested
- ○ Not sure/Prefer not to answer

How would you rate your current risk of an undiagnosed STI like HIV, syphilis, chlamydia, or gonorrhoea?

- High Risk
- Medium Risk (Average Risk)
- Low Risk
- Unsure/Prefer not to answer

Pre_anxiety

Anxiety questions before the website

In this section, you will see six questions (01 to 06) asking about your current feelings of anxiety. For each question, please select the option that best indicates how you feel right now, at this moment. Do not spend too much time on any one question. Your immediate response is what is needed.

Please answer every question, even if you are unsure of the best answer.

	Not at all	Somewhat	Moderately	Very much
01. I feel calm.	○	○	○	○
02. I feel tense.	○	○	○	○
03. I feel upset.	○	○	○	○
04. I feel relaxed.	○	○	○	○
05. I feel content.	○	○	○	○
06. I feel worried.	○	○	○	○

This is the end of part I: Pre-survey. Click continue, and you will be taken to the website to check your risk of HIV/STIs.

- Continue to MySTIRisk
- Do not continue, and stop the survey

Powered by Qualtrics

Part II: MySTIRisk Website

Part III

Post Introduction

Part 3: Post-website Survey

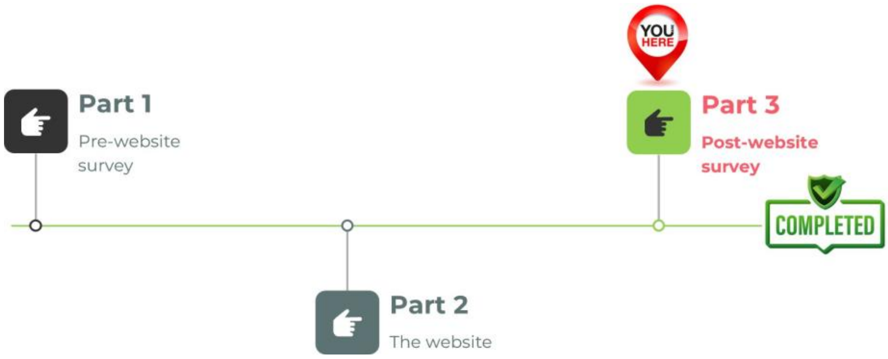

Great! You have now got to Part 3: the final part of the survey.

Block 3

Part 3: Post-website Survey

When answering, focus on your feelings **AFTER viewing your risk report**.

How high was the risk level on the MySTIRisk website?

- ○ High
- ○ Average
- ○ Unsure/Prefer not to answer

How likely are you to get tested for HIV/STIs in the next three months?

- ○ Extremely unlikely
- ○ Somewhat unlikely
- ○ Neither likely nor unlikely
- ○ Somewhat likely
- ○ Extremely likely
- ○ Unsure/Prefer not to answer

Post_anxiety

Anxiety questions after using the website

In this section, you will see **six questions (01 to 06)** asking about your **current feelings of anxiety related to your risk report after using MySTIRisk website**. For each question, please select the option that best indicates how you feel right now, at this moment. Do not spend too much time on any one question. Your immediate response is what is needed.

Please answer every question, even if unsure of the best answer.

	Not at all	Somewhat	Moderately	Very much
01. I feel calm.	O	O	O	O
02. I feel tense.	O	O	O	O
03. I feel upset.	O	O	O	O
04. I feel relaxed.	O	O	O	O
05. I feel content.	O	O	O	O
06. I feel worried.	O	O	O	O

post_high_anxiety_msg (logic – appear only if post-website STAI-6 scores > 14)

Thank you for your time today.

Your score shows you are feeling quite anxious now. We suggest you talk to a professional soon. They can help provide coping strategies and support.

We have counselling available at the Melbourne Sexual Health Centre on 03 9341 6200. Ask for results and information line and say you are involved in the MySTIRisk study. They will arrange a counsellor's appointment for you.

We appreciate you taking the time to complete this survey. Your responses, even though we cannot continue the full study today, will help us better understand and address the anxiety that many people experience. We wish you all the best.

Please click the button below to continue.

O Continue

MySTIRisk Questions

How easy or difficult was MySTIRisk to use and understand?

- Very difficult
- Somewhat difficult
- Neutral
- Somewhat easy
- Very easy
- Unsure/Prefer not to answer

Was the information provided by MySTIRisk clear and understandable?

- Very Unclear
- Unclear
- Neutral
- Clear
- Very Clear
- Unsure/Prefer not to answer

Do you feel your MySTIRisk results will lead you to change any sexual behaviors?

- Yes
- No
- Unsure/Prefer not to answer

Would you recommend MySTIRisk to friends/partners?

- Yes
- No
- Unsure/Prefer not to answer

Voucher

Would you like to enter the prize draw to win a $50 gift voucher?
If "yes", a link where you will be prompted to enter your email address will appear at the end of the survey.

- Yes
- No

Powered by Qualtrics
