# Supplementary material for: Anxiety responses and testing intentions among gay and bisexual men using an AI-powered HIV/STI risk assessment tool: a quasi-experimental study
Source: BMC Public Health. 2025 Nov 18;25:4028. doi: 10.1186/s12889-025-25064-2 (PMC12625431; doi:10.1186/s12889-025-25064-2)
Supplement: Supplementary file 2 — Supplementary Material 2. [file 12889_2025_25064_MOESM2_ESM.pdf]

Part I

Introduction

Introduction

Thank you for your interest in participating in this research. You received a link to this survey via an SMS text message because you previously consented to getting health-related messages from the Melbourne Sexual Health Centre.

We are developing a tool to help members of the public assess their risk of acquiring HIV and other sexually transmitted infections (STIs), named “MySTIRisk”. In this study, we aim to evaluate the effects of the MySTIRisk website on user’s perceived risk, and anxiety related to HIV/STIs.

You will be randomly assigned to either use the MySTIRisk website or view the MSHC webpage providing sexual health information. The surveys before and after will allow us to assess changes in anxiety levels. The pre-procedure survey will collect background information. The post-procedure survey will ask about changes in emotions, specifically anxiety, after viewing your assigned website.

We would like to invite you to participate in this short survey, which will take around 15 minutes of your time to complete.

You do not have to participate in this survey if you don't want to. This survey will not collect any information that can identify you. Your responses to this survey will remain confidential to the MSHC research team.

This study is approved by the Alfred Hospital Ethics Committee (741/23). Please read the participant information sheet by clicking HERE.

If you agree to participate, please click the "Agree" button below.
If you don't agree to participate, please click the "Disagree" button below.

- ○ Agree
- ○ Disagree

Block 3

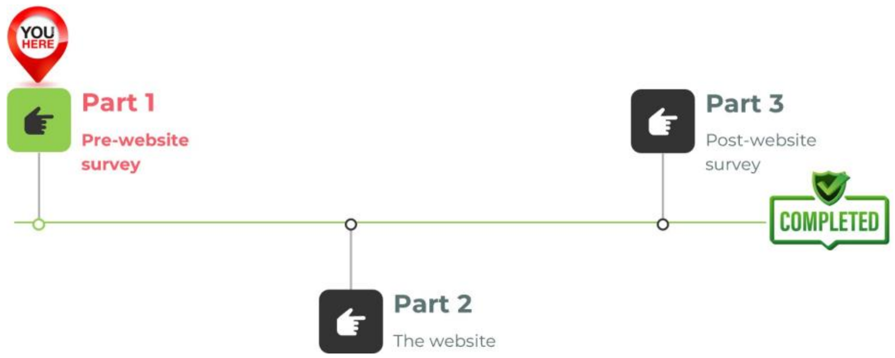

This survey has 3 parts:

Part 1: Pre-website Questions
In this first part, we ask you things about yourself and 6 questions about how you are feeling.

Part 2: The website

Next, you will either use the MySTIRisk website or view a standard sexual health webpage. Please read carefully. Please read carefully.

Part 3: Post-Website Questions

Finally, you'll answer questions about how the website made you feel, especially any anxiety.

Please answer honestly - we are interested in your opinions and feelings. Your responses are anonymous.

Demographic Questions

Part 1: Pre-website questions

What sex were you assigned at birth?

- ○ Male
- ○ Female
- ○ Intersex
- ○ Don’t know/ Prefer not to say

Do you consider yourself to be:

- ○ Lesbian / Gay / Homosexual
- ○ Bisexual
- ○ Straight/Heterosexual
- ○ Queer
- ○ ________ Different identity, Please specify:
- ○ Prefer not to say

How old are you? (write number of years)

____

Where were you born?

- Australia
- Other country
- Not sure/Prefer not to answer

How long have you been in Australia?

Less than 1 year

- Less than 5 years
- 5 years or longer
- Not sure/Prefer not to answer

What is the highest level of education you have completed?

- Postgraduate level
- Bachelor level
- Diploma level
- Certificate level
- High school
- Primary school
- __________ Other (Please Specify)
- Not sure/Prefer not to answer

What is your current employment status? (Tick all that apply]

- Student
- Full-time employment or self-employed
- Part-time /casual employment
- Retired
- Unemployed or not working
- Unable to work
- __________ Other (Please specify)
- Not sure/Prefer not to answer

Have you ever been tested for sexually transmissible infections such as HIV, syphilis, gonorrhoea or syphilis?

- Yes
- No
- Not sure/Prefer not to answer

Have you ever been diagnosed with sexually transmissible infections such as HIV, syphilis, gonorrhoea or syphilis?

- Yes
- No
- Not sure/Prefer not to answer

When was your last HIV/STI test?

- Within the past 6 months
- Within the past year
- 1-2 years ago
- More than 2 years ago
- Never tested
- Not sure/Prefer not to answer

How would you rate your current risk of an undiagnosed STI like HIV, syphilis, chlamydia, or gonorrhoea?

- ○ High Risk
- ○ Medium Risk (Average Risk)
- ○ Low Risk
- ○ Unsure/Prefer not to answer

Pre_anxiety

Anxiety questions before the website

In this section, you will see **six questions** (**01 to 06**) asking about your **current feelings of anxiety**. For each question, please select the option that best indicates how you feel **right now**, **at this moment**. Do not spend too much time on any one question. Your immediate response is what is needed.

Please answer every question, even if unsure of the best answer.

	Not at all	Somewhat	Moderately	Very much
01. I feel calm.	○	○	○	○
02. I feel tense.	○	○	○	○
03. I feel upset.	○	○	○	○
04. I feel relaxed.	○	○	○	○
05. I feel content.	○	○	○	○
06. I feel worried.	○	○	○	○

Pre_end

You have **completed Part 1** of this study, which included the demographic and anxiety measurement questions. Thank you!

Please click "continue" to proceed to view the webpage sexual health information provided by the Melbourne Sexual Health Centre.

- ○ Continue

Part II: The Control Webpage

Control Webpage

Part 2: The webpage providing the sexual health information by MSHC

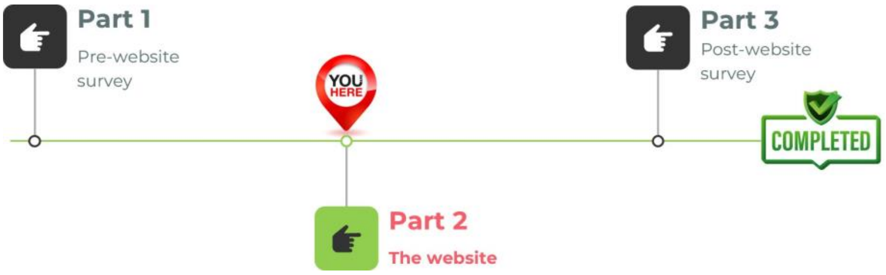

In this section, you will see the screenshots of the webpage providing sexual health information by the Melbourne Sexual Health Centre. Please read the information carefully. You will also see two videos, which only lasted over one minute. Please make sure you watched those videos as well.

If you are using your phone, please ZOOM IN to read the information.

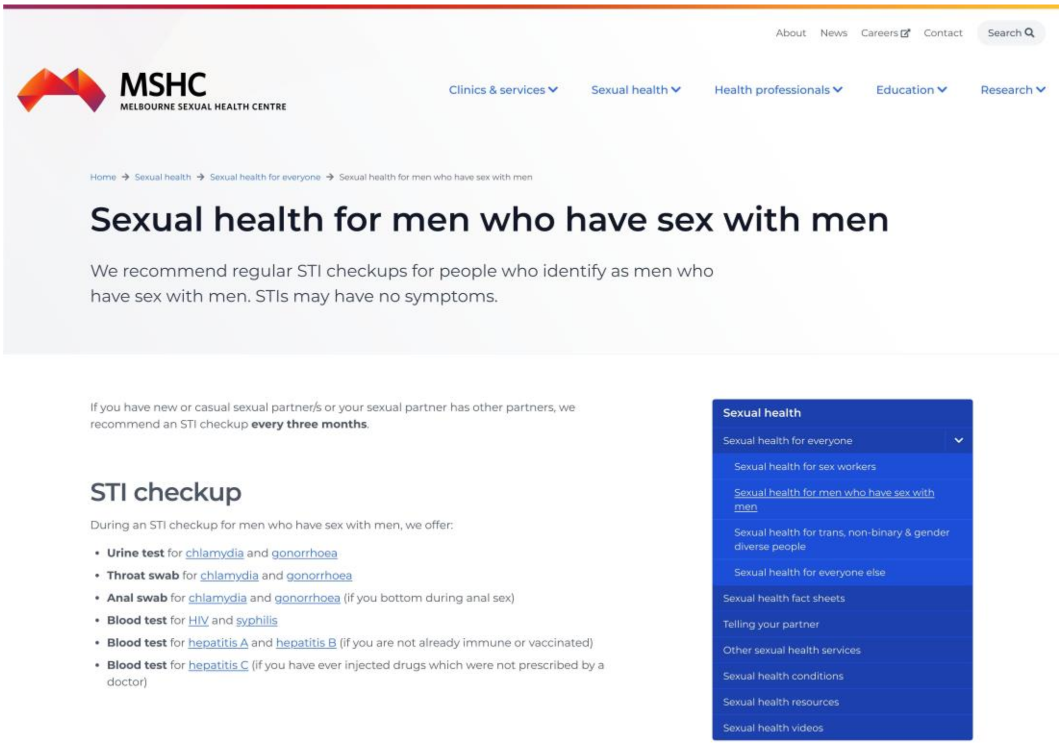

About News Careers Contact Search

MSHC
MELBOURNE SEXUAL HEALTH CENTRE

Clinics & services | Sexual health | Health professionals | Education | Research

Home → Sexual health → Sexual health for everyone → Sexual health for men who have sex with men

Sexual health for men who have sex with men

We recommend regular STI checkups for people who identify as men who have sex with men. STIs may have no symptoms.

If you have new or casual sexual partner/s or your sexual partner has other partners, we recommend an STI checkup **every three months**.

STI checkup

During an STI checkup for men who have sex with men, we offer:

- **Urine test** for chlamydia and gonorrhoea
- **Throat swab** for chlamydia and gonorrhoea
- **Anal swab** for chlamydia and gonorrhoea (if you bottom during anal sex)
- **Blood test** for HIV and syphilis
- **Blood test** for hepatitis A and hepatitis B (if you are not already immune or vaccinated)
- **Blood test** for hepatitis C (if you have ever injected drugs which were not prescribed by a doctor)

Sexual health
- Sexual health for everyone
  - Sexual health for sex workers
  - Sexual health for men who have sex with men
  - Sexual health for trans, non-binary & gender diverse people
  - Sexual health for everyone else
- Sexual health fact sheets
- Telling your partner
- Other sexual health services
- Sexual health conditions
- Sexual health resources
- Sexual health videos

If you are using your phone, please ZOOM IN to read the information.

HIV window period

When you have a HIV test, it will not tell us anything about any possible HIV exposures from risky sex in the 6 weeks before the day of the HIV test. This 6 week period is called the window period. If you have had risky sex in this 6 week period, you will have to retest. A HIV blood test done during the window period may show a person does not have HIV when in fact they do have HIV.

Related links

- PEP fact sheet
- Get PEP
- Alfred Health: Victorian NPEP Service
- PrEP fact sheet
- Alfred Health: Victorian PrEP Service
- PrEP Clinic
- PrEP'D for change

Had a HIV risk?

Post exposure prophylaxis (PEP) is a HIV medication which is taken within 72 hours of possible exposure to HIV infection and is taken for 28 days. In most cases, PEP can stop HIV from establishing itself in the body and can prevent you from becoming HIV positive

To be at risk of HIV you need to have had risky contact with a person who has HIV.

Risky contact includes:

- penetrative sex
- sharing a needle/syringe
- other sex which involved blood

To get PEP, you must come to our walk in and wait clinic >

Want to get on PrEP?

PrEP is highly effective in preventing HIV when taken consistently every day or on-demand under the advice of your doctor.

PrEP is suitable for you if you are at high risk of HIV infection. For example:

- if you are a man who has sex with men without using a condom
- if you have a sexual partner who has HIV infection and is not on treatment, or is at high risk of getting HIV

PrEP can be prescribed by any GP.

You can find a GP in your area at Prep Access Now.

If you are using your phone, please ZOOM IN to read the information.

Safe sex practices

Safe sex practices can reduce your risk for STIs.

Use:

- condoms for penetrative sex. Always check the use-by date, as old condoms can break easily.
- dental dam (a thin plastic barrier) for oral sex.
- water-based lubricant to reduce the chance of the condom or dam breaking. Don't use Vaseline® or massage oil as this can weaken the condom or dam.
- new condom or dam each time you have sex (even if you or your partner didn't ejaculate). Never wash out a condom and use it again.

Blood is a high risk fluid for HIV transmission. If you are practicing sexual activities that involve blood, use gloves. Gloves are important for protecting both participants. Surgical gloves are best.

Activities could include:

- fisting
- S&M
- piercing

If you are using drugs, sharing injecting or snorting equipment increases your risk of blood borne viruses. Use your own clean equipment and do not share equipment to minimise your risk.

https://www.youtube.com/watch?v=rmpwoLRDF38&ab_channel=EndingHIV

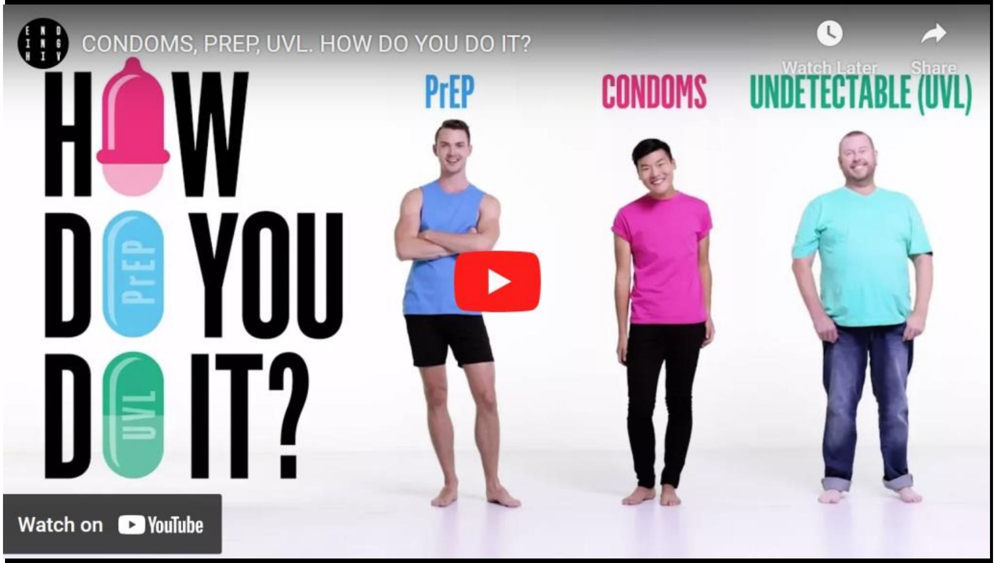

0:00

https://www.youtube.com/watch?v=bkuE2ojLwWM&ab_channel=
ThorneHarbourHealth

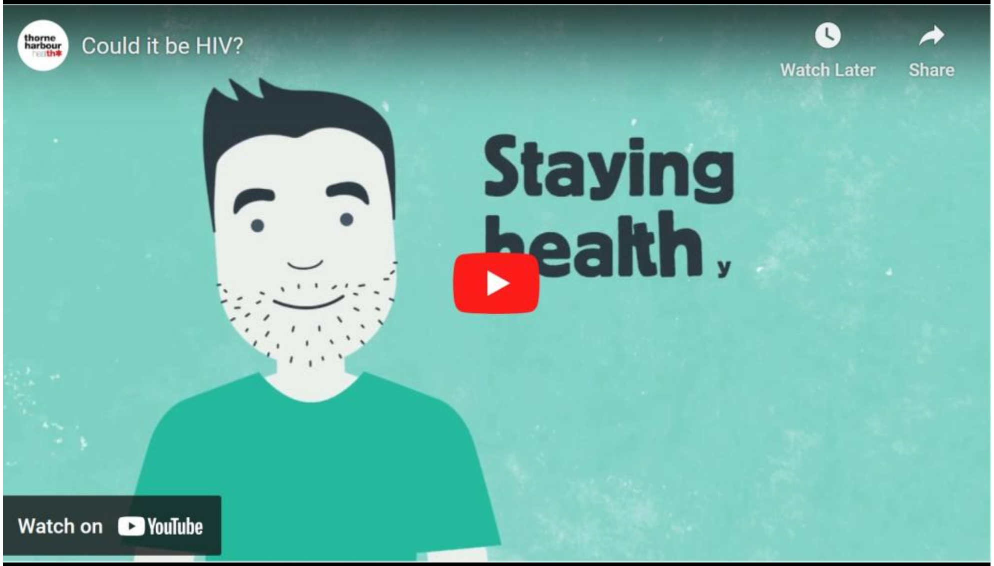

0:00

You have completed Part 2 of this study, which included viewing the webpage sexual health information provided by the Melbourne Sexual Health Centre. Thank you! Please click "**continue**" to proceed to the Part 3: Post-website questions.

○ Continue

Part III

Post Introduction

Part 3: Post-website Questionnaires

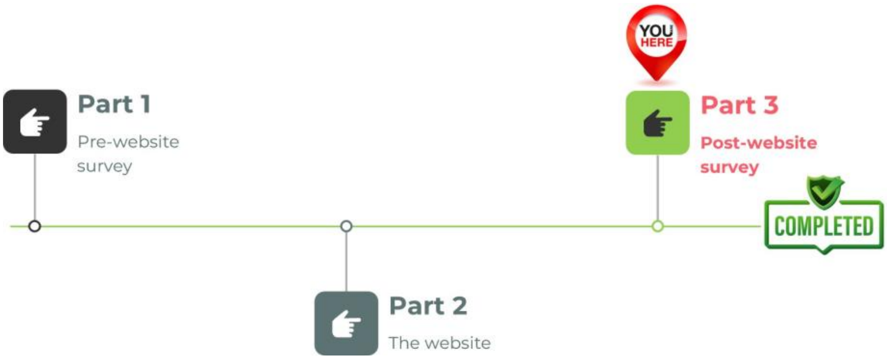

Great! You have now got to Part 3: the final part of the survey.
Click "Continue" to proceed.

- ○ Continue

Block 3

Part 3: Post-website Questionnaires

Now that you have viewed the webpage providing sexual health information, please complete the following questions again. When answering, focus specifically on your feelings AFTER viewing the website. Try to isolate the effect of seeing the risk report on your anxiety levels.

DO NOT CONSIDER OTHER FACTORS OR PAST FEELINGS OF ANXIETY. Concentrate only on the anxiety or related emotions you felt immediately after viewing your risk report and any anxious feelings still lingering after seeing the report.

Please read each question carefully and indicate the answer that best reflects your POST-REPORT ANXIETY LEVELS. Do not overthink your responses.
There are no right or wrong answers; we are simply interested in understanding the effects of personalised sexual health risk reports on people's temporary emotions.

After viewing the website, how likely are you to get tested for HIV/STIs in the next three months?

- ○ Extremely unlikely
- ○ Somewhat unlikely
- ○ Neither likely nor unlikely
- ○ Somewhat likely
- ○ Extremely likely
- ○ Unsure/Prefer not to answer

Post_anxiety

Anxiety questions after using the website

In this section, you will see six questions (01 to 06) asking about your current feelings of anxiety related to your risk report after viewing the website. For each question, please select the option that best indicates how you feel right now, at this moment. Do not spend too much time on any one question. Your immediate response is what is needed.

Please answer every question, even if unsure of the best answer.

	Not at all	Somewhat	Moderately	Very much
01. I feel calm.	○	○	○	○
02. I feel tense.	○	○	○	○
03. I feel upset.	○	○	○	○
04. I feel relaxed.	○	○	○	○
05. I feel content.	○	○	○	○
06. I feel worried.	○	○	○	○

post_high_anxiety_msg (logic – appears only if post-survey STAI-6 scores > 14)

Thank you for your time today.

Based on your responses, we encourage you to talk to someone about the anxious feelings you may be experiencing lately. Please know that it's very common to feel stressed or overwhelmed at times. Talking to a professional can help provide coping strategies and support.

For assistance, please contact the counselling centre at the Melbourne Sexual Health Centre at 03 9341 6200. The counsellors are available to help provide completely confidential and judgement-free support.

We appreciate you taking the time to complete this survey. Your responses, even though we cannot continue the full study today, will help us better understand and address the anxiety that many people experience. We wish you all the best.

Please click the button below to continue.

○ Continue

Voucher

Would you like to enter the prize draw to win a $50 gift voucher?
If "yes", a link where you will be prompted to enter your email address will appear at the end of the survey.

○ Yes
○ No

Powered by Qualtrics
