# Supplementary material for: Anxiety responses and testing intentions among gay and bisexual men using an AI-powered HIV/STI risk assessment tool: a quasi-experimental study
Source: BMC Public Health. 2025 Nov 18;25:4028. doi: 10.1186/s12889-025-25064-2 (PMC12625431; doi:10.1186/s12889-025-25064-2)
Supplement: Supplementary file 3 — Supplementary Material 3. [file 12889_2025_25064_MOESM3_ESM.docx]

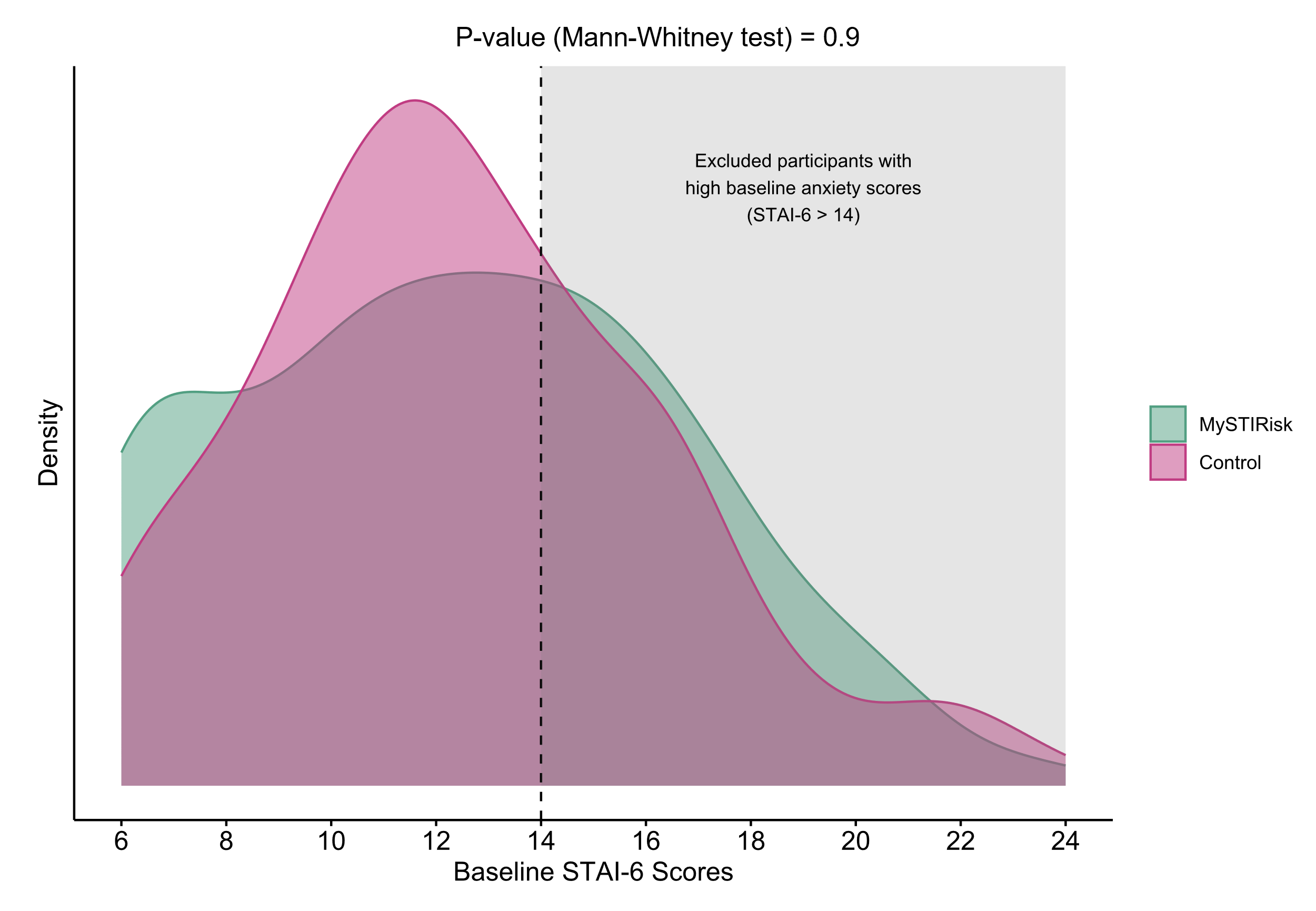


Figure S1. Distribution of Baseline STAI-6 scores for 530 Participants Before Excluding Those with High Baseline Anxiety Scores (STAI-6 >14) and Incomplete Responses


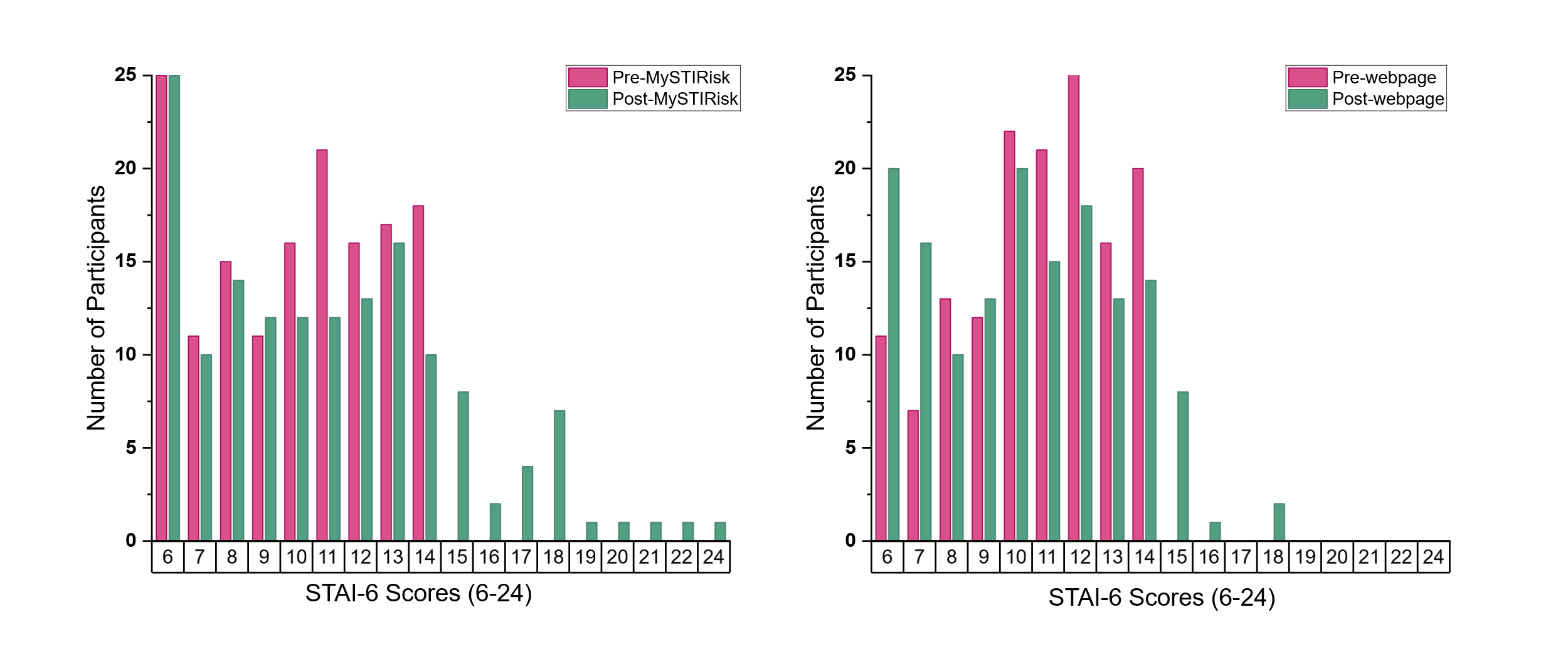


Figure S2. Distribution of STAI-6 scores Before and After Website Use by Group

Table S1. The Six-item State-Trait Anxiety Inventory (STAI-6) Scale*

|  | **Not at all** | **Somewhat** | **Moderately** | **Very Much** |
| --- | --- | --- | --- | --- |
| (1) I feel calm | 1 | 2 | 3 | 4 |
| (2) I am tense | 1 | 2 | 3 | 4 |
| (3) I feel upset | 1 | 2 | 3 | 4 |
| (4) I am relaxed | 1 | 2 | 3 | 4 |
| (5) I feel content | 1 | 2 | 3 | 4 |
| (6) I am worried | 1 | 2 | 3 | 4 |

*Note: Each item is scored on a 4-point Likert scale (1-4). Items 1, 4, and 5 are reverse-coded during analysis. Total scores range from 6 to 24, with higher scores indicating greater anxiety. STAI-6 is a validated, shortened version of the original 20-item STAI-State scale.

Table S2. Testing History and Risk Perception for 530 Participants Before Excluding Those with High Baseline Anxiety Scores (STAI-6 >14) and Incomplete Responses

|  | **Total (N = 530)** | **No Pre-existing Anxiety [STAI-6 ≤14] (n = 366)** | **High Anxiety [STAI-6 > 14] (n = 164)** | **P-value** |
| --- | --- | --- | --- | --- |
| **Previous HIV/STI Diagnosis, n (%)** | | | | |
| Yes | 372 | 272 (74.3%) | 100 (61.0%) | 0.002 |
| No | 151 | 92 (25.1%) | 59 (36.0%) |  |
| Don't know/Prefer not to answer | 7 | 2 (0.5%) | 5 (3.0%) |  |
| **Last HIV/STI Test, n (%)** | | | | |
| Never tested | 24 | 12 (3.3%) | 12 (7.3%) | 0.2 |
| Within the past 6 months | 451 | 316 (86.3%) | 135 (82.3%) |  |
| Within the past year | 19 | 14 (3.8%) | 5 (3.0%) |  |
| 1-2 years ago | 22 | 14 (3.8%) | 8 (4.9%) |  |
| More than 2 years ago | 13 | 10 (2.7%) | 3 (1.8%) |  |
| Don't know/Prefer not to answer | 1 | - | 1 (0.6%) |  |
| **Self-Perceived STI Risk, n (%)** | | | | |
| High Risk | 81 | 55 (15.0%) | 26 (15.9%) | 0.5 |
| Medium Risk (Average Risk) | 192 | 133 (36.3%) | 59 (36.0%) |  |
| Low Risk | 242 | 165 (45.1%) | 77 (47.0%) |  |
| Don't know/Prefer not to answer | 15 | 13 (3.6%) | 2 (1.2%) |  |

Table S3. User Feedback on MySTIRisk Website (n=150)

| **Feedback Domain** | **MySTIRisk (n=150)** |
| --- | --- |
| **Ease of Use, n (%)** |  |
| Very/Somewhat easy | 138 (92.0) |
| Neutral | 10 (6.7) |
| Very difficult | 1 (0.7) |
| Unsure/Prefer not to answer | 1 (0.7) |
| **Information Clarity, n (%)** |  |
| Very/Clear | 136 (90.7) |
| Neutral | 9 (6.0) |
| Very Unclear/Unclear | 3 (2.0) |
| Unsure/Prefer not to answer | 2 (1.3) |
| **Anticipate Changing their Sexual Behaviour, n (%)** | |
| Yes | 49 (32.7) |
| No | 86 (57.3) |
| Unsure/Prefer not to answer | 15 (10.0) |
| **Would Recommend to Others, n (%)** | |
| Yes | 109 (72.7) |
| No | 20 (13.3) |
| Unsure/Prefer not to answer | 21 (14.0) |
