# Supplementary material for: Anxiety responses and testing intentions among gay and bisexual men using an AI-powered HIV/STI risk assessment tool: a quasi-experimental study
Source: BMC Public Health. 2025 Nov 18;25:4028. doi: 10.1186/s12889-025-25064-2 (PMC12625431; doi:10.1186/s12889-025-25064-2)
Supplement: Supplementary file 4 — Supplementary Material 4. [file 12889_2025_25064_MOESM4_ESM.pdf]

Appendix A: MySTIRisk

Version 2 (archived content as of November 2024)

Welcome screen content

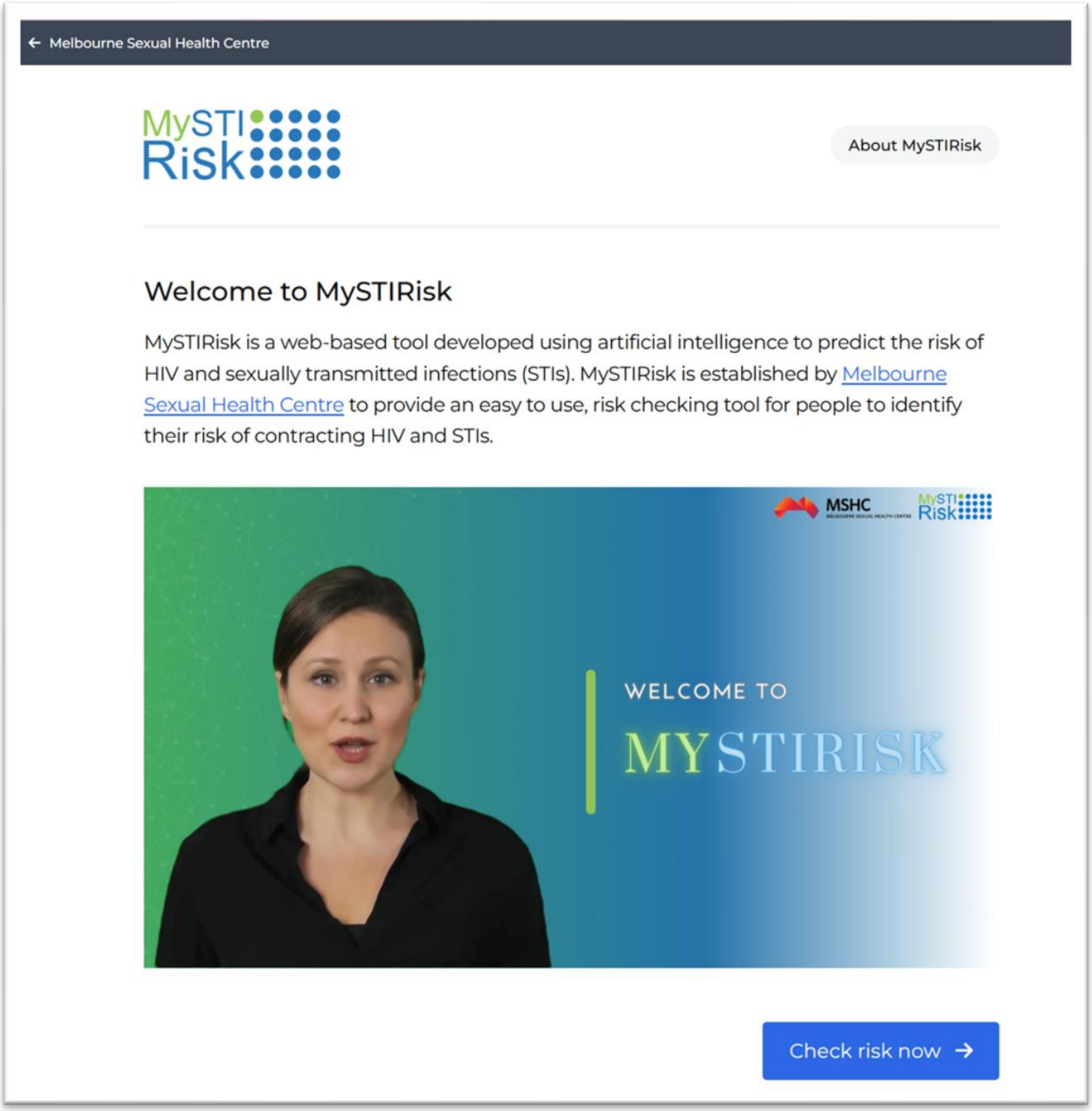

Example of questions

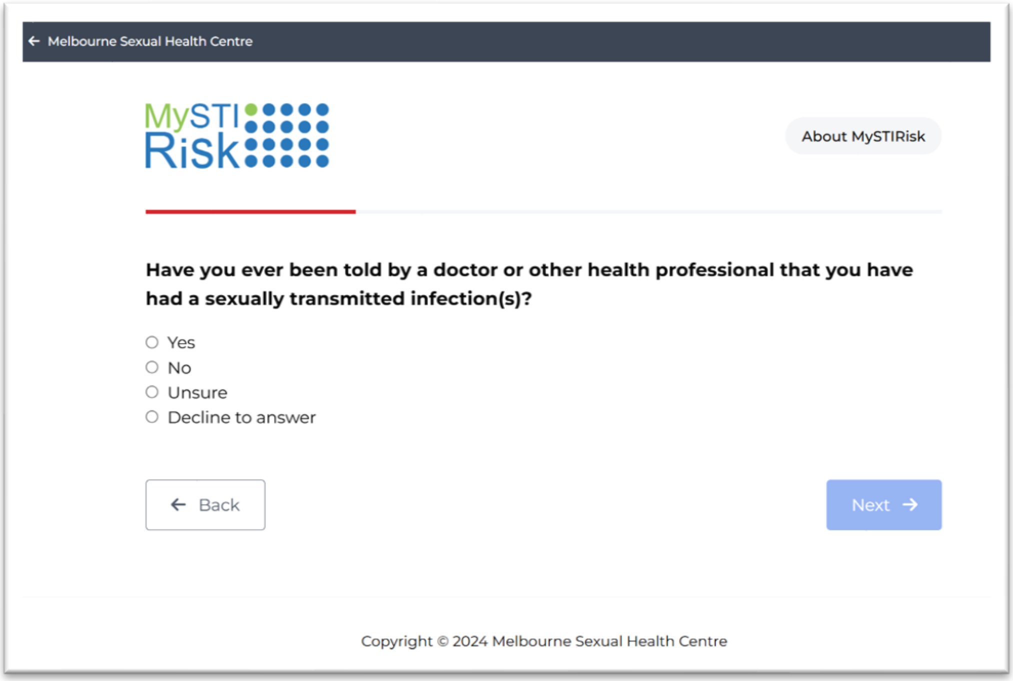

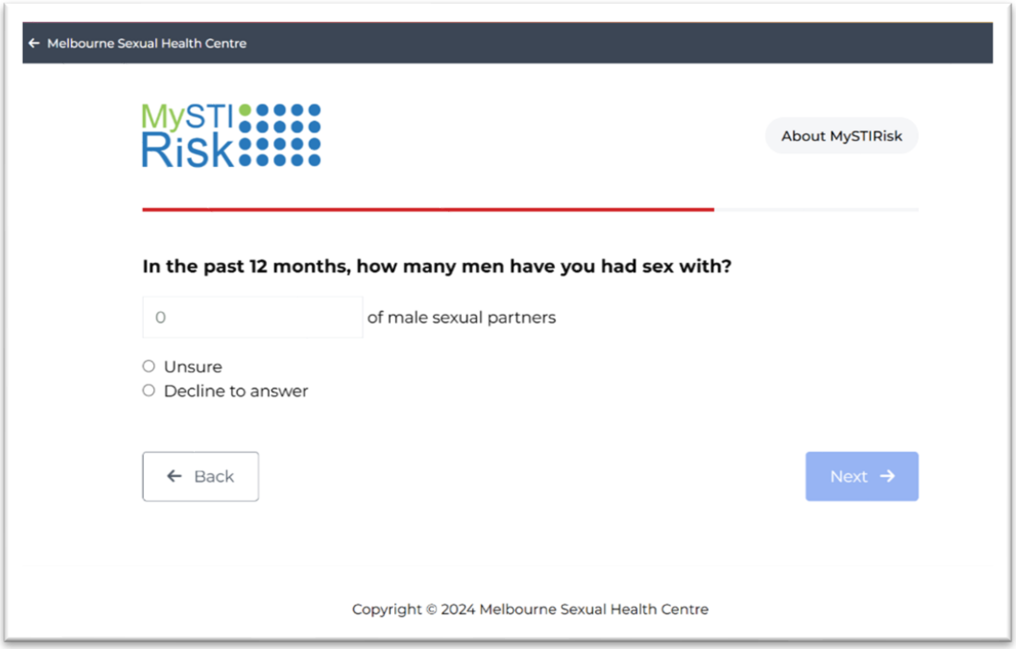

An example of MySTIRisk Report

← Melbourne Sexual Health Centre

MySTI Risk

About MySTIRisk

STI Risk Report

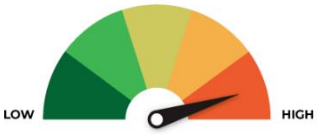

High risk

Based on your responses, you have been identified as **high risk** of STIs.
We recommend that you **get tested** as soon as possible.

We have generated the risk of the four infections for a person with the same answers that you gave but who attended Melbourne Sexual Health Centre. These risks are below.

Risk of HIV

- **Their risk of HIV is high.**
- At present, in a group of **1000 people**, **4** will have HIV.
- Over the next 12 months, in a group of **1000 people**, **less than 1** are likely to catch HIV.

Risk of Syphilis

- **Their risk of syphilis is high.**
- At present, in a group of **100 people**, **11** will have syphilis.
- Over the next 12 months, in a group of **100 people**, **4** are likely to catch syphilis.

Risk of Gonorrhoea

- **Their risk of gonorrhoea is high.**
- At present, in a group of **100 people**, **27** will have gonorrhoea.
- Over the next 12 months, in a group of **100 people**, **12** are likely to catch gonorrhoea.

Risk of Chlamydia

- **Their risk of chlamydia is high.**
- At present, in a group of **100 people**, **15** will have chlamydia.
- Over the next 12 months, in a group of **100 people**, **10** are likely to catch chlamydia.

This report can be downloaded in PDF format on the recommendations page.

Recommendations →

Recommendations

MySTI Risk

About MySTIRisk

Reduce my risk recommendations

You can reduce your risk or harm from these infections by:

- Having regular tests to detect infections early (reduce complications)
- Using condoms for vaginal or anal sex (reduces HIV or STI risk 90-95%)
- Taking PrEP if you are at high risk of acquiring HIV (see https://www.pan.org.au/)(reduce HIV risk by 99%)

In addition, a person can reduce the harm from these infections by testing

Benefits of testing

- Prevents complications from the infections by early treatment
- Prevents unknowing transmitting the infection to others

Without timely treatment, you may develop complications from the infections including

- Infertility (untreated chlamydia or gonorrhoea)
- Chronic pain (untreated chlamydia or gonorrhoea)
- Loss of hearing or sight (untreated syphilis)
- Cancer (untreated HIV)
- Higher risk of complications from untreated HIV infection such as cancer or infections (treatment of HIV is safe and easy and prevents these complications)

Where to go

If you consider yourself at risk, we recommend you take your results to your General Practitioner. To find General Practitioners click here.

It is essential that you see a doctor if you have any genital, anal, or physical symptoms, or if you think you could have an infection. If one of your sexual partners has told you that they have an infection, let your doctor know as it may assist with your care.

These recommendations are only intended to be a guide and may not necessarily suit your individual circumstances. If you have been tested for STIs recently, you may not need to be retested again.

Further information

For your interest and information, sexual health fact sheets can be found here. Treatment guidelines for sexually transmitted infections can be found here. Partner notification information can be found here.

This report has been generated by the MySTIRisk web application on the Melbourne Sexual Health Centre website. We applied machine-learning approach using retrospective data from thousands of clients attending our service, to generate the most likely and other possible diagnoses. These suggested diagnoses do not substitute professional medical advice or consultations with healthcare professionals.

Download my report (pdf)

← Back

Start again →
