# Supplementary material for: Anxiety responses and testing intentions among gay and bisexual men using an AI-powered HIV/STI risk assessment tool: a quasi-experimental study
Source: BMC Public Health. 2025 Nov 18;25:4028. doi: 10.1186/s12889-025-25064-2 (PMC12625431; doi:10.1186/s12889-025-25064-2)
Supplement: Supplementary file 5 — Supplementary Material 5. [file 12889_2025_25064_MOESM5_ESM.pdf]

Sexual health for men who have sex with men

We recommend regular STI checkups for people who identify as men who have sex with men. STIs may have no symptoms.

In this section

-- Sexual health for men who have sex with men

If you have new or casual sexual partner/s or your sexual partner has other partners, we recommend an STI checkup **every three months**.

STI checkup

During an STI checkup for men who have sex with men, we offer:

- **Urine test** for chlamydia and gonorrhoea
- **Throat swab** for chlamydia and gonorrhoea
- **Anal swab** for chlamydia and gonorrhoea (if you bottom during anal sex)
- **Blood test** for HIV and syphilis
- **Blood test** for hepatitis A and hepatitis B (if you are not already immune or vaccinated)
- **Blood test** for hepatitis C (if you have ever injected drugs which were not prescribed by a doctor)

HIV window period

When you have a HIV test, it will not tell us anything about any possible HIV exposures from risky sex in the 6 weeks before the day of the HIV test. This 6 week period is called the window period. If you have had risky sex in this 6 week period, you will have to retest. A HIV blood test done during the window period may show a person does not have HIV when in fact they do have HIV.

Had a HIV risk?

Post exposure prophylaxis (PEP) is a HIV medication which is taken within 72 hours of possible exposure to HIV infection and is taken for 28 days. In most cases, PEP can stop HIV from establishing itself in the body and can prevent you from becoming HIV positive

To be at risk of HIV you need to have had risky contact with a person who has HIV.

Risky contact includes:

- penetrative sex
- sharing a needle/syringe
- other sex which involved blood

Want to get on PrEP?

PrEP is highly effective in preventing HIV when taken consistently every day or on-demand under the advice of your doctor.

PrEP is suitable for you if you are at high risk of HIV infection. For example:

- if you are a man who has sex with men without using a condom
- if you have a sexual partner who has HIV infection and is not on treatment, or is at high risk of getting HIV

PrEP can be prescribed by any GP.

You can find a GP in your area at Prep Access Now.

Safe sex practices

Safe sex practices can reduce your risk for STIs.

Use:

- condoms for penetrative sex. Always check the use-by date, as old condoms can break easily.
- dental dam (a thin plastic barrier) for oral sex.
- water-based lubricant to reduce the chance of the condom or dam breaking. Don’t use Vaseline® or massage oil as this can weaken the condom or dam.
- new condom or dam each time you have sex (even if you or your partner didn’t ejaculate). Never wash out a condom and use it again.

Blood is a high risk fluid for HIV transmission. If you are practicing sexual activities that involve blood, use gloves. Gloves are important for protecting both participants. Surgical gloves are best.

Activities could include:

- fisting
- S&M
- piercing

If you are using drugs, sharing injecting or snorting equipment increases your risk of blood borne viruses. Use your own clean equipment and do not share equipment to minimise your risk.

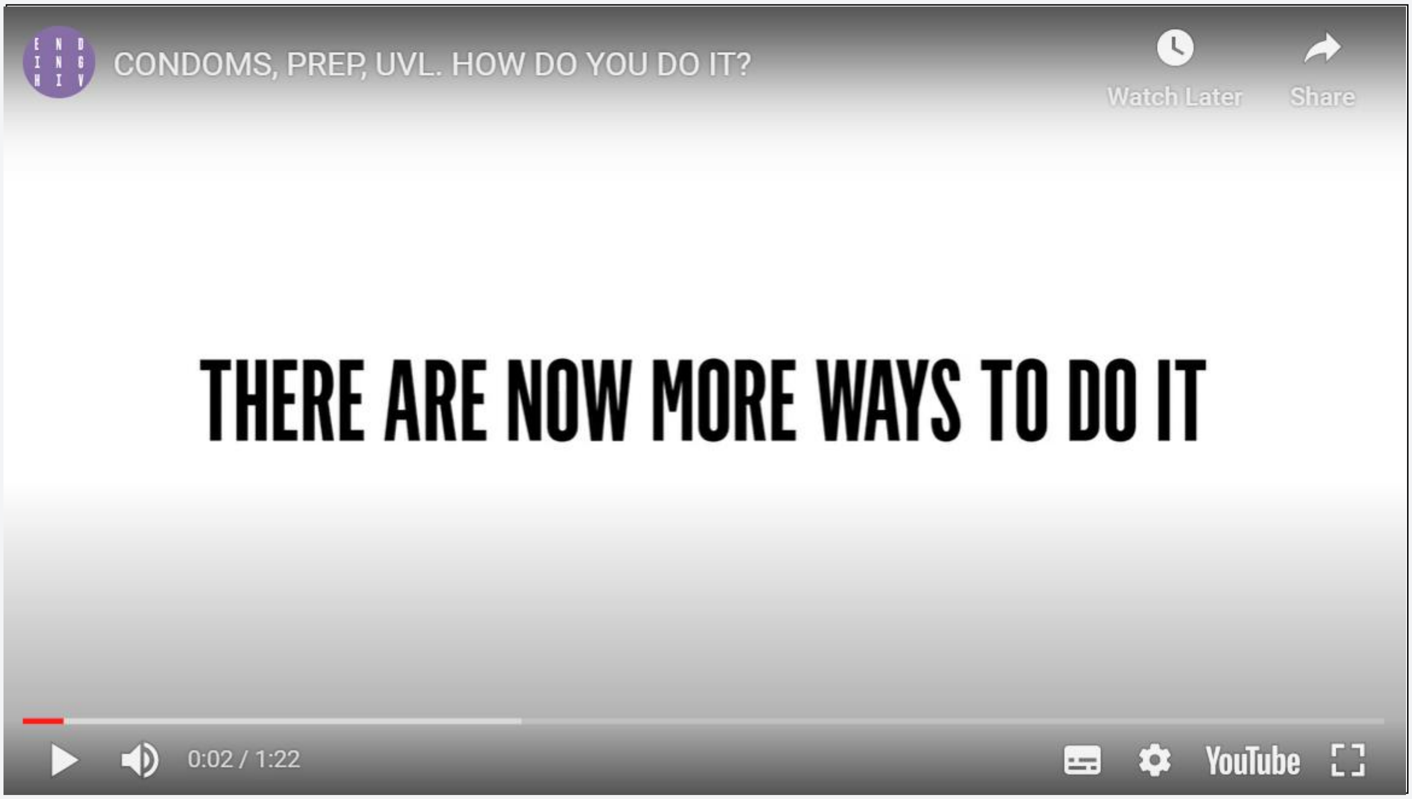

Video Link: https://youtu.be/rmpwoLRDF38

Condoms, PrEP, UVL. How do you do it?

There are now more ways to do it. Protecting yourself from HIV that is. Meet Tom, Dick and Harry. These guys all like a good time, but they stay safe in different ways.

Credit: [Ending HIV]

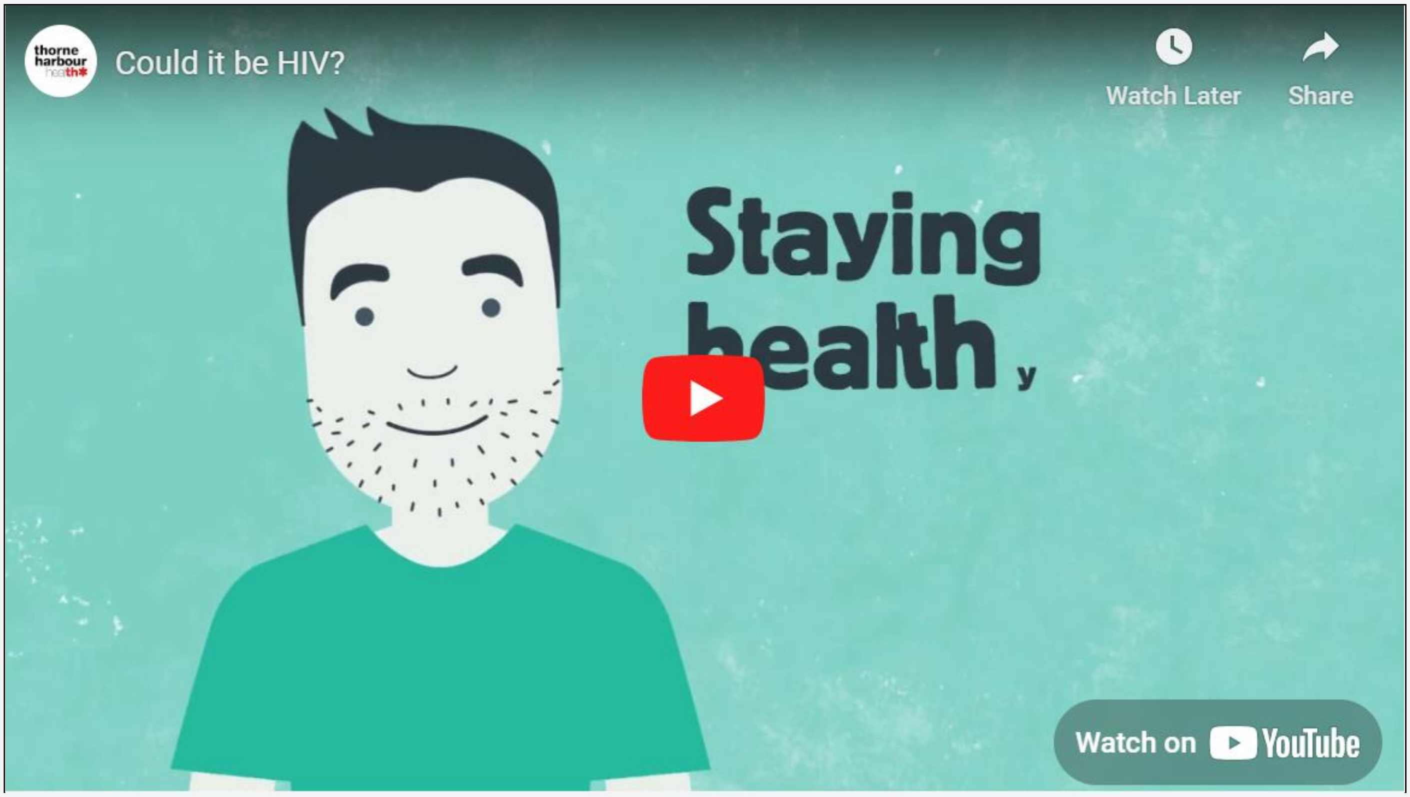

Video Link: https://youtu.be/bkuE2ojLwWM

Could it be HIV?

Seroconversion illness is the illness that occurs when your body comes into contact with HIV. This short video provides 3 quick things to know about HIV seroconversion illness.

Credit: Thorne Harbour Health

Related links

PEP fact sheet

Get PEP

Alfred Health: Victorian NPEP Service

PrEP fact sheet

Alfred Health: Victorian PrEP Service

PrEP Clinic

PrEP'D for change

Centre opening hours

Mon-Fri 8.30am-5pm
Closed Victorian public holidays

Phone
03 9341 6200

Freecall
1800 032 017
Call us for free from a fixed line or mobile phone outside Melbourne metropolitan area

National Relay Service
03 9341 6200
Visit the website

Visit
580 Swanston Street
Carlton 3053 Victoria

Get directions

We acknowledge the people of the Kulin Nation who are the custodians of the land and waters, and pay our respects to Elders past and present.
We welcome all cultures, nationalities and religions. Being inclusive and providing equitable healthcare is our commitment.

Privacy statement
Disclaimer
Accessibility
Website feedback

Copyright © 2024 Melbourne Sexual Health Centre

MSHC
MELBOURNE SEXUAL HEALTH CENTRE
Part of
AlfredHealth
